# Supplementary material for: Small cohort of patients with epilepsy showed increased activity on Facebook before sudden unexpected death
Source: Epilepsy Behav. Author manuscript; Available in PMC 2023 Oct 18. (PMC10582639; doi:10.1016/j.yebeh.2022.108580)
Supplement: Supplementary Material [file NIHMS1933350-supplement-Supplementary_Material.pdf]

Small cohort of patients with epilepsy showed increased activity on *Facebook* before sudden unexpected death

Ian B. Wood[a], Rion Brattig Correia[b,c,a], Wendy R. Miller[d] and Luis M. Rocha[e,a,b]

[a]Center for Social and Biomedical Complexity, Luddy School of Informatics, Computing & Engineering, Indiana University, Bloomington, IN 47408 USA

[b]Instituto Gulbenkian de Ciência, Oeiras 2780-156, Portugal

[c]CAPES Foundation, Ministry of Education of Brazil, Brasília, DF, Brazil

[d]School of Nursing, Indiana University, Indianapolis, IN 46202 USA

[e]Department of Systems Science and Industrial Engineering, Binghamton University, Binghamton, NY 13902

Supplemental Material

Subject	μ_1	n_1	μ_2	n_2	*intercept*	$time_{coef}$	$time_{se}$	$time_p$	θ	θ_{se}
2	12.431	2162	34.413	109	2.520	1.018	0.086	**1.197e-32**	1.373	0.043
1	9.592	1547	17.889	54	2.261	0.623	0.135	**4.146e-06**	1.113	0.043
8	12.070	2185	18.375	16	2.491	0.420	0.241	0.081	1.153	0.036
6	5.252	717	7.304	23	1.659	0.330	0.143	**0.021**	3.136	0.269
10	13.983	1147	23.571	7	2.638	0.522	0.264	**0.048**	2.254	0.105
3	11.125	834	4.100	10	2.409	-0.998	0.312	**0.001**	1.385	0.072

Table S1: Statistics from a Negative Binomial Regression on Word Count per Post. μ_1 and n_1 correspond to the mean word count and number of posts before the last two months, while μ_2 and n_2 correspond to the mean word count and number of posts during the last two months before SUDEP. Also included are the *intercept* of the regression, the coefficient on the last month indicator variable $time_{coef}$, its standard error $time_{se}$, the p-value of the coefficient $time_p$, and the dispersion parameter θ with its standard error θ_{se}.

A negative binomial model is often used to model over-dispersed count data, i.e. when the variance is considerably larger than the mean [S1]. Here a negative binomial model is estimated through a generalized linear regression with log link function on word count per post over a dummy variable representing whether the post's word count occurs during the last month. The significance of the time-indicator dummy variable estimates the significance of the change in the last month over all other posts. As shown in Table S1 we see significant increases in the word count per post for four subjects at $p < 0.05$. The table is ordered according to the rank product of the number of posts before and during the last two months preceding SUDEP, and the two with the greatest number of posts in both periods by rank product are also the two with the greatest increase in word count, subjects 2 and 1, with two additional subjects showing significant increases, subject 6 and 10. There are five subjects with decreases in word count per post, with subject 11 and subject 3 with significant decreases.

An alternative formulation is to examine word count per day rather than per post. Perhaps some subjects additionally start posting short posts with increased frequency during periods of stress. However, many days contain zero posts, thus zero words, for most subjects. We can model this with a zero-inflated negative binomial model that also estimates a probability that no words will be posted [S1, S2]. As shown in Table S2 we see that subject 2 and 1 still have significant increases in word count per day (columns $time_{coef}$ and $time_p$) and both are significantly more likely to post during the last 2 months (columns $0_{time_{coef}}$ and 0_{time_p},

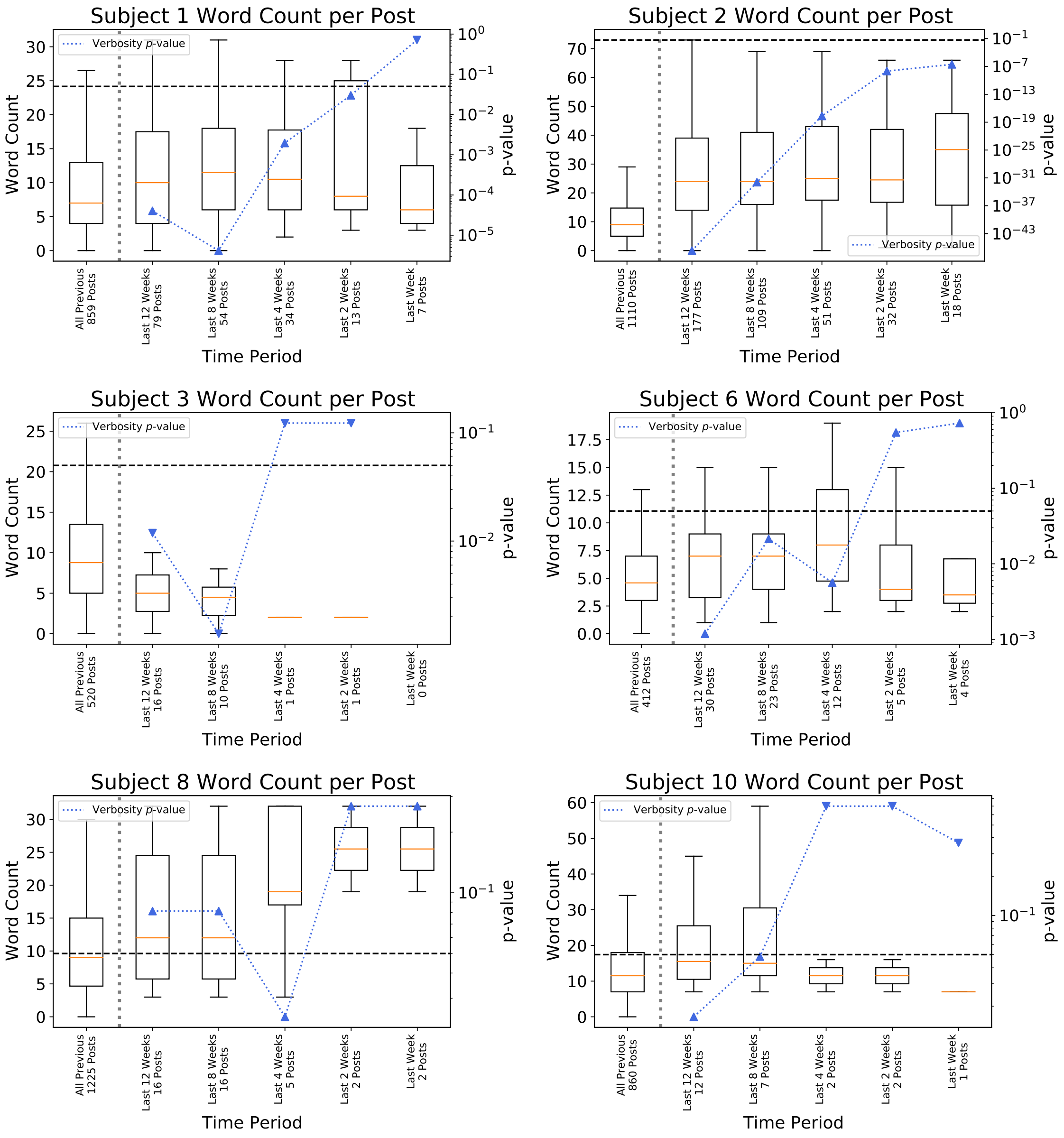

Figure S1: Subject verbosity per post over different epochs. Difference between word count per post in the period immediately preceding SUDEP compared to word count per post during earlier posting periods. Different selections of the time window for the last posting period are displayed on the x-axis. The box plot on the far left represents all posts before the 12 weeks preceding SUDEP. The blue line represents the p-value of the time coefficient for the negative binomial regression. The direction of the arrow represents the sign of the coefficient, up indicates an increase in wordcount during the period preceding SUDEP and down indicates a decrease. The horizontal black line represents p-value=0.05.

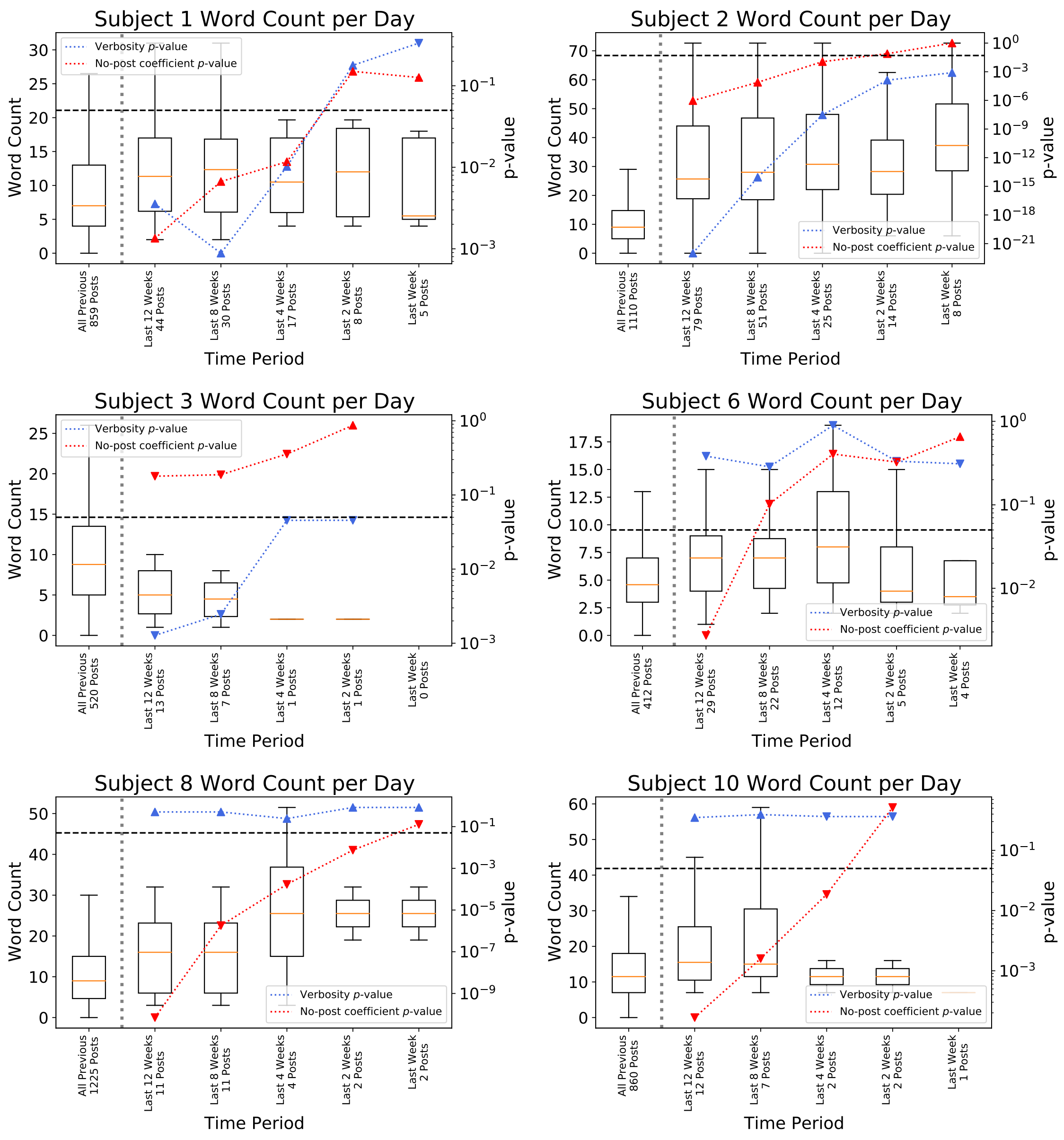

Figure S2: Subject verbosity per day over different epochs. Difference between word count per day in the period immediately preceding SUDEP compared to word count per day during earlier posting periods. Different selections of the time window for the last posting period are displayed on the x-axis. The box plot on the far left represents all posts before the 12 weeks preceding SUDEP. The blue line represents the p-value of the word count time coefficient for the zero-inflated negative binomial regression. The direction of the blue triangle represents the sign of the coefficient, up indicates an increase in wordcount during the period preceding SUDEP and down indicates a decrease. The red line represents the p-value of the zero post time coefficient of the regression, with red triangles representing whether there is an increase in the likelihood of any post on a day (up) or a decrease (down). The horizontal black line represents $p = 0.05$.

note the negative coefficient corresponds to a lower probability of having no posts on a given day). Subjects 8 and 10 are significantly less likely to post during the last two months. Subject 11, however, is significantly more likely to post during the last two months, although with significantly fewer words per day. Subject 3 is seen to have a significant drop in word count per day. Additionally, subjects 7 and 5 are significantly more likely to post in the last two months, but with non-significant changes in word count. This view of the posting behavior also reveals interesting patterns but is not particularly more informative than the negative binomial model per post.

Subject	$intercept$	$time_{coef}$	$time_{se}$	$time_p$	$0_{intercept}$	$0_{time_{coef}}$	0_{time_p}
2	3.114	1.185	0.153	**8.802e-15**	-0.275	-1.854	**7.779e-05**
1	2.821	0.621	0.187	**8.828e-04**	0.586	-0.755	**0.007**
8	3.028	0.213	0.318	0.503	-0.261	1.637	**1.802e-06**
6	2.170	-0.213	0.199	0.285	-0.183	0.490	0.102
10	2.914	0.240	0.281	0.393	0.513	1.295	**0.002**
3	2.829	-1.234	0.408	**0.002**	0.991	0.571	0.187

Table S2: Statistics of a Zero-Inflated Negative Binomial Regression on word count per day. This is similar to Table S1, but models the word count per day rather than per post, with the addition of a logistic regression model representing the likelihood of no post at all. Included are the $intercept$ of the regression, the coefficient on the last month indicator variable $time_{coef}$, its standard error $time_{se}$, the p-value of the coefficient $time_p$. Additionally, parameters of the logistic regression on no-post probabilities are shown: the intercept $0_{intercept}$, the coefficient on the time indicator $0_{time_{coef}}$ and the significance of this coefficient 0_{time_p}.

References

S1. Zero-Inflated Negative Binomial Regression R Data Analysis Examples. 2018 Aug. Available from: https://stats.idre.ucla.edu/r/dae/zinb/

S2. Zeileis A, Kleiber C, and Jackman S. Regression Models for Count Data in R. Journal of Statistical Software 2008; 27. Available from: http://www.jstatsoft.org/v27/i08/
